# Longitudinal evaluation examining implementation and sustainment of an opioid overdose education and naloxone distribution among veterans who are unstably housed

**DOI:** 10.1186/s43058-025-00764-3

**Published:** 2025-08-06

**Authors:** Sarah J. Javier, Amanda M. Midboe, Taryn Perez, Angela M. Kyrish, Madeleine Golding, Hannah Cheng, Kenneth Bruemmer, Danica Bogicevic

**Affiliations:** 1 VA Health Systems Research Center for Innovation to Implementation (Ci2i), VA Palo Alto Healthcare System, 795 Willow Road, Menlo Park, CA 94025 USA; 2 Stanford University School of Medicine, 291 Campus Drive, Stanford, CA 94305 USA; 3 Department of Public Health Sciences, School of Medicine, University of California Davis, One Shields Avenue, Davis, CA 95616 USA; 4 VA Health Systems Research Center for Health Optimization and Implementation Research (CHOIR), VA Bedford Healthcare System, 200 Springs Road (152), Building 70, Bedford, MA 01730 USA; 5 VHA Homeless Programs Office, Department of Veterans Affairs, 810 Vermont Avenue, NW Washington, DC 20420 USA

**Keywords:** Harm reduction, Homelessness, Implementation, Naloxone, Opioid, Overdose, Permanent supportive housing, Qualitative, Quality improvement, Sustainability, Veteran

## Abstract

**Background:**

Rigorous implementation evaluations are needed to understand factors that influence implementation and sustainability of evidence-based interventions across contexts. In this study, we conducted a longitudinal, multi-methods, multi-site evaluation guided by the Dynamic Sustainability Framework (DSF). This evaluation focused on implementation of the Homeless Overdose Prevention—Expansion (HOPE), an opioid overdose education and naloxone distribution (OEND) trial in a permanent supportive housing program in the Veterans Health Administration (VA).

**Methods:**

We used a multi-methods study design comprised of qualitative interviews and completion of a three-item survey. Semi-structured interviews were completed with Department of Housing and Urban Development-VA Supportive Housing (HUD-VASH) staff, site leaders, and site prescribers at four VA healthcare systems in the Western United States. Interviews were conducted at three timepoints: pre-implementation, implementation, and sustainment. Site staff also completed the Provider REport of Sustainment Scale (PRESS) during sustainment to provide more context for our interpretation of results. We analyzed interview data using rapid directed content analysis guided by DSF constructs and analyzed PRESS using descriptive statistics.

**Results:**

We conducted 96 interviews with 67 unique individuals. Six determinants influenced the reciprocal fit of the intervention, practice setting, and ecological system across our study: (1) OEND for unstably housed veterans; (2) Staffing shortages and competing demands; (3) Training-related concerns; (4) Supervisor and leadership buy-in at site; (5) Social workers’ scope of practice; and (6) Cultural climate and saliency of OEND initiatives. Both planned and unplanned implementation strategies were optimized by different actors (i.e., the implementation team, target practitioners) in response to evolving determinants at all three DSF levels to maintain overall fit. Approaches taken to optimize fit depended on longitudinal data collection and evaluation of determinants at each phase.

**Conclusions:**

Our approach is a theoretically driven example of capturing important determinants of implementation and sustainment of OEND in a high-risk setting. Despite the uniqueness of our study setting, our approach is generalizable and has the potential to promote sustainability of other public health interventions.

**Supplementary Information:**

The online version contains supplementary material available at 10.1186/s43058-025-00764-3.

Contributions to the Literature

- This paper demonstrates a novel, theoretically informed approach to evaluating implementation and sustainment of an evidence-based, harm reduction intervention longitudinally.
- Findings demonstrate that the intervention, context, and ecological system, constructs from the Dynamic Sustainability Framework, interact reciprocally over time when applied to the implementation and sustainment of an opioid overdose education and naloxone distribution intervention in a setting serving unstably housed individuals.
- Iterative, longitudinal data collection and analysis across different stages of implementation is important for optimization of fit of evidence-based practices in a given clinical setting.

## Introduction

Opioid-related overdose deaths have increased nearly fourfold over the past decade [1]. These deaths are caused by both prescription opioids and opioids obtained illicitly [2, 3]. Fentanyl, a synthetic opioid that is 50 times stronger than heroin, is increasingly present in non-opioid drugs including stimulants. Illicitly manufactured fentanyl has contributed substantially to overdose deaths in the U.S. and is the primary driver of recent waves of the opioid epidemic [3, 4].

Opioid Overdose Education and Naloxone Distribution (OEND) is an evidence-based, federally-endorsed harm reduction intervention aimed at preventing opioid overdose deaths [5, 6]. OEND comprises opioid overdose prevention education and distribution of naloxone, a lifesaving opioid antagonist [7]. Naloxone commonly comes in the form of a nasal spray and is easily administered by laypeople and medical personnel alike, with a nearly 90% success rate in reversing opioid overdose [8].

Despite clear benefits of OEND, there are many barriers to its widespread dissemination that extend to populations at highest risk for overdose. Unhoused and unstably housed individuals are more likely to die from opioid overdose than individuals in the general population [9–11]. These individuals face increased exposure to environmental stressors that exacerbate chances of overdose and encounter unique challenges in accessing healthcare [11–14]. Yet, many unhoused individuals are never offered OEND. Midboe and colleagues found that less than 25% of veterans receiving VA care who had a diagnosis of opioid use disorder received a prescription for naloxone in the year following entry into a VA homeless program [15]. This statistic is alarming given that national clinical practice guidelines and state and federal policies recommend that naloxone is offered to all individuals at increased risk for opioid overdose [16, 17].

The purpose of this longitudinal qualitative evaluation was to rigorously evaluate factors relevant to implementation and sustainment of OEND in a VA homeless program setting. Because there is a critical need to address sustainment of evidence-based interventions in line with a framework [18], we used the Dynamic Sustainability Framework (DSF) as a guide for our evaluation [19]. The DSF posits that intervention sustainment depends on the reciprocal fit between an evidence-based intervention, a practice setting, and the ecological system. Thus, it is important to adapt both interventions and implementation strategies over time to maintain optimal fit across these three domains and to continue producing intended outcomes. This study utilized the DSF to examine dynamic factors at the ecological, practice-setting, and individual levels that influence implementation and sustainment of an OEND intervention in a Veterans Health Administration (VA) homeless program. To our knowledge, this is the first paper to apply the DSF to a multisite study using a longitudinal, multi-methods approach.

## Methods

This is a longitudinal, multisite study across three time-points: pre-implementation, implementation, and sustainment. We relied primarily on qualitative methods, excepting use of a quantitative measure during the sustainment phase. This evaluation was embedded within a hybrid type 3 effectiveness-implementation trial [18], with the primary aim to evaluate education and audit and feedback strategies for implementation of OEND in a VA homeless program setting. The OEND clinical team included a case manager and a prescriber, with the case manager providing overdose education (OE) and the prescriber distributing naloxone (ND). Almost all case managers within the implementation setting were social workers; thus, we refer to them as social workers throughout the remainder of the paper. The clinical setting is the U.S. Department of Housing and Urban Development – VA Supportive Housing (HUD-VASH) program settings in the Sierra Pacific region of the United States. HUD-VASH’s primary goal is to provide housing and case management to unhoused veterans [20]. There are four implementation sites included in this evaluation. Included sites were below the national average for at-risk HUD-VASH veterans receiving naloxone and were selected in partnership with VISN 21 Homeless Coordinators. This study was deemed as quality improvement, with a non-human subjects research exemption obtained from the Institutional Review Board of record.

## Qualitative methods

### Interview guides

Semi-structured interview guides included domains from the DSF for each of the three implementation phases (See Appendices A-C). For instance, for the DSF “intervention” domain, we developed pre-implementation interview questions around components of OEND and who should deliver components of OEND (See Appendix A). All three interview guides were reviewed by the larger HOPE team for clarity and precision. This review included pilot interview sessions with a VA social worker with OEND implementation experience who provided feedback to improve the guide.

### Data collection

**Fig. 1:**
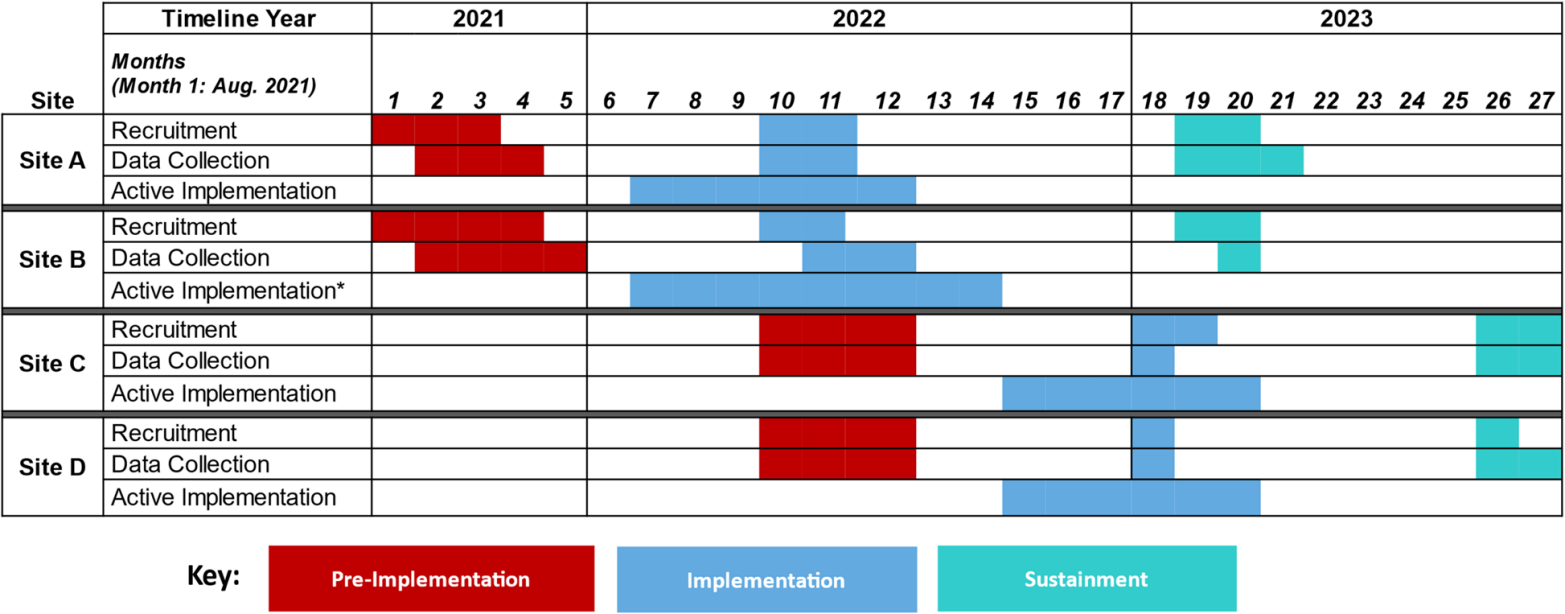
Data collection timeline across four sites *Note: Site B had eight months of active implementation

Pre-implementation interviews were initiated before implementation. Implementation-focused interviews occurred at one time-point during the six-month implementation period. During implementation, site personnel attended one-hour training sessions once per month, for a total of six sessions per VA Healthcare System (VAHCS). The exception was Site B, which received two additional trainings due to a significant implementation barrier that was eventually resolved but impacted the implementation timeline. Figure 1 reflects the recruitment and data collection timeline.

We interviewed HUD-VASH program managers, supervisors, social workers, and prescribers at pre-implementation at four VAHCSs. At implementation and sustainment phases, we interviewed HUD-VASH program coordinators and prescribers and selectively sampled social workers who had low and high OEND outreach during the first three months of implementation.

Participants were recruited via encrypted e-mail. Responsive individuals were scheduled for an individual interview using Microsoft Teams. During interview sessions, one qualitatively trained interviewer (AK, HC, MG, SJ, or TP) and a designated notetaker (AK, HC, MG, or TP) were present, along with the interview participant. Interviews were audio and/or video-recorded with the participants’ verbal consent and notetakers took detailed notes for all interviewees using a predesigned template. Interview notes were the primary source of data for analysis, but we also used Microsoft Teams transcriptions and recordings to verify information from the notes.

### Analytic approach

We utilized a rapid directed content analysis based on the DSF to analyze interview data [21]. We first developed tailored definitions for each DSF construct, relying on the Consolidated Framework for Implementation Research (CFIR) to define any construct not defined in the DSF (Table 1) [22].

**Table 1:** Adapted DSF definitions

DSF Domain	DSF Constructs	Adapted DSF Definitions
Intervention: often includes a set of individual components chosen for their ability to effect behavior change	Components	Individual components of the intervention chosen for their ability to reduce opioid overdose risk, i.e., opioid overdose education and naloxone distribution (DSF)
	Practitioners	Innovation deliverers; i.e., individuals who are directly or indirectly delivering OEND to veterans in HUD-VASH (example: OE delivered by social workers, ND delivered by site prescribers) (CFIR)
	Outcomes	Targeted, patient-centered outcomes that OEND should generate as a result of its use in HUD-VASH (DSF)
	Delivery Platform	The platform by which OEND is delivered to veterans in HUD-VASH (e.g., face-to-face, phone outreach, etc.) (DSF)
Practice Setting/Context: The DSF anchors the ultimate benefit of the intervention in terms of its ability to fit within a practice setting, typically a clinical or community setting	Staffing	Work infrastructure that can support OEND implementation; organization of tasks and responsibilities within and between HUD-VASH staff and teams, and general staffing levels that can support OEND implementation (CFIR)
	Information systems	Information technology structure; the technological systems for tele-communication, electronic documentation, and data storage, management, reporting, and analysis that support OEND implementation in the practice setting. HOPE utilizes two technological information systems: 1) a provider-facing electronic health record (EHR) on which referral and treatment notes are organized; and 2) a case-finding dashboard, which allows users to filter information and identify OEND-eligible veterans, their assigned social workers, and other site information (CFIR)
	Organizational culture	Shared values, beliefs and norms about opioids, OEND, and substance use in the practice setting (CFIR)
	Training	Access to knowledge and information about OEND; there is guidance and/or training (e.g., training slides related to resources, OEND calls, etc.) accessible to HUD-VASH staff that helps them to implement OEND (CFIR)
	Leadership/Supervision	High-level leaders: Individuals with a high level of authority within and outside of VA operations including state and national HUD-VASH leadership and HPO, including key decision-makers, executive leaders, or directors (CFIR) Mid-level leaders: Site leadership who have a moderate level of authority and are responsible for overseeing programs. May or may not be embedded in HUD-VASH but they have authority when it comes to implementation of OEND programs (e.g., Chief of Social Work at Site B, HUD-VASH program managers across sites) (CFIR) Supervisors: HUD-VASH supervisors under HUD-VASH program managers who supervise individual teamlets (CFIR)
Ecological Systems: At a third level, the DSF identifies the ecological system as an additional driver of the successful implementation and sustainability of an intervention	Other practice settings	Equivalent to outer setting, or the VA site at which a HUD-VASH program exists. Could also include the local community around the VA site, as some Veterans may get Narcan within community settings outside of VA. (CFIR)
	Policy	VA, state, and/or federal legislation that either supports or hinders OEND implementation within HUD-VASH. (CFIR)
	Regulations	Regulations, professional group guidelines and recommendations, or accreditation standards that support or hinder OEND implementation in HUD-VASH. This domain could also include changing regulations that have the potential to impact implementation. (CFIR)
	Market forces	Competing with and/or imitating local, regional, state, and national OEND initiatives drives or hinders OEND implementation. (CFIR)
	Population characteristics	Characteristics of unhoused or unstably housed Veterans who are at risk for opioid overdose. (CFIR)

The qualitative team (AK, HC, MG, SJ, TP) met prior to analyzing interview data to develop and refine the codebook, then coded one to three interviews together to standardize procedures. The rest of the interviews were completed individually, with the group meeting iteratively to discuss any concerns and maintain consensus. After coding completion, codes were transferred into a matrix organized by interviewee position and site to identify themes in the data as they pertained to our tailored DSF constructs. We conducted data quality checks throughout the analytic process [23] and completed the Consolidated criteria for Reporting Qualitative Research checklist (See Appendix D).

### Quantitative methods

We administered an adapted version of the 3-item Provider Report of Sustainment Scale (PRESS) to all interviewees during sustainment to provide context for our qualitative findings. PRESS is measured on a Likert-type scale from 0 = not at all to 4 = to a very great extent (Table 2). We used descriptive statistics (e.g., mean, range, standard deviation) to analyze PRESS results.

**Table 2:** Provider REport of Sustainment Scale (PRESS) items adapted for the OEND intervention

INSTRUCTIONS: The following questions ask about OEND at your VA medical center. Please indicate the extent to which you agree with the following items:
1. Staff use OEND as much as possible when appropriate
2. Staff continue to use OEND throughout changing circumstances
3. OEND is a routine part of our practice

Response options include 0 = not at all, 1 = to a slight extent, 2 = to a moderate extent, 3 = to a great extent, and 4 = to a very great extent

## Results

**Fig. 2:**
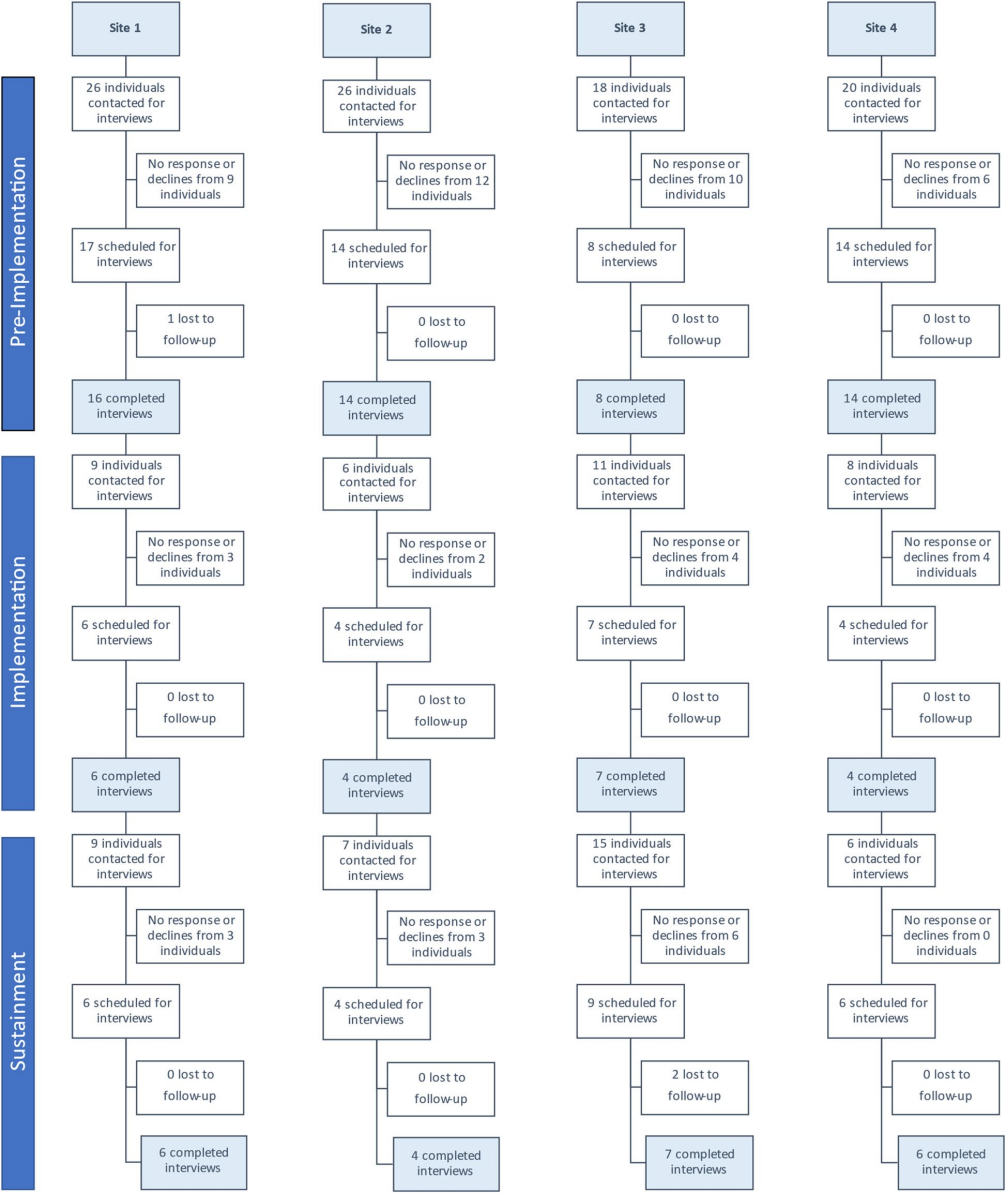
Recruitment and data collection across all time-points

We conducted 96 interviews with 67 unique individuals across three time-points. Figure 2 indicates which participants agreed to or declined an interview during each phase.

All interviews lasted between 20–60 min. One interview from the sustainment period was a combined interview with two prescribers. Tables 3 and 4 present characteristics of interviewees.

**Table 3:** Characteristics of interviewees across timepoints

Timepoint	Site A (*n* = 28)	Site B (*n* = 22)	Site C (*n* = 22)	Site D (*n* = 24)
*Pre-Implementation (n* = *51)*	*16*	*14*	*7*	*14*
HUD-VASH program manager	1	2	1	1
HUD-VASH supervisor	4	3	2	0
HUD-VASH social worker	6	3	2	3
Site prescriber	1	3	1	2
Site leadership	0	2	1	3
Other position				
SUD Specialist	3	0	0	1
Peer Support Specialist	1	0	0	0
Occupational Therapist	0	1	0	0
Non-HUD-VASH social worker	0	0	0	2
Social work intern	0	0	0	1
Nurse	0	0	0	1
*Implementation (n* = *22)*	*6*	*4*	*8*	*4*
HUD-VASH program manager	1	1	1	1
HUD-VASH supervisor	2	3	0	0
HUD-VASH social worker	1	0	5	0
Site prescriber	1	0	2	1
Other position				
SUD Specialist	1	0	0	0
Non-HUD-VASH supervisor	0	0	0	1
Social work intern	0	0	0	1
*Sustainment (n* = *23)*	*6*	*4*	*7*	*6*
HUD-VASH program manager	1	1	1	1
HUD-VASH supervisor	1	1	3	0
HUD-VASH social worker	0	0	2	3
Site prescriber	3	1	1	2
Site leadership	0	1	0	0
Other position				
SUD Specialist	1	0	0	0

18 individuals were interviewed at multiple timepoints

**Table 4:** Characteristics of interviewees (*N* = 67)

Characteristic	Site A (*n* = 20)	Site B (*n* = 14)	Site C (*n* = 18)	Site D (*n* = 15)
Years at VA *M(SD)*	9.11(6.04)	6.89(5.66)	8.73(6.72)	9.27(8.93)
Years in HUD-VASH *M(SD)* (*n* = 49 interviewees)	7.03(4.80)	4.32(2.57)	3.58(2.62)	4.67(3.85)
Position (*n*)				
HUD-VASH program manager (*n* = 5)	1	2	1	1
HUD-VASH supervisor (*n* = 11)	4	3	4	0
HUD-VASH social worker (*n* = 22)	7	3	9	3
Site prescriber (*n* = 11)	4	3	2	2
Site leadership (*n* = 4)	0	2	0	2
Other position (*n* = 14)	4	1	2	7

**Fig. 3:**
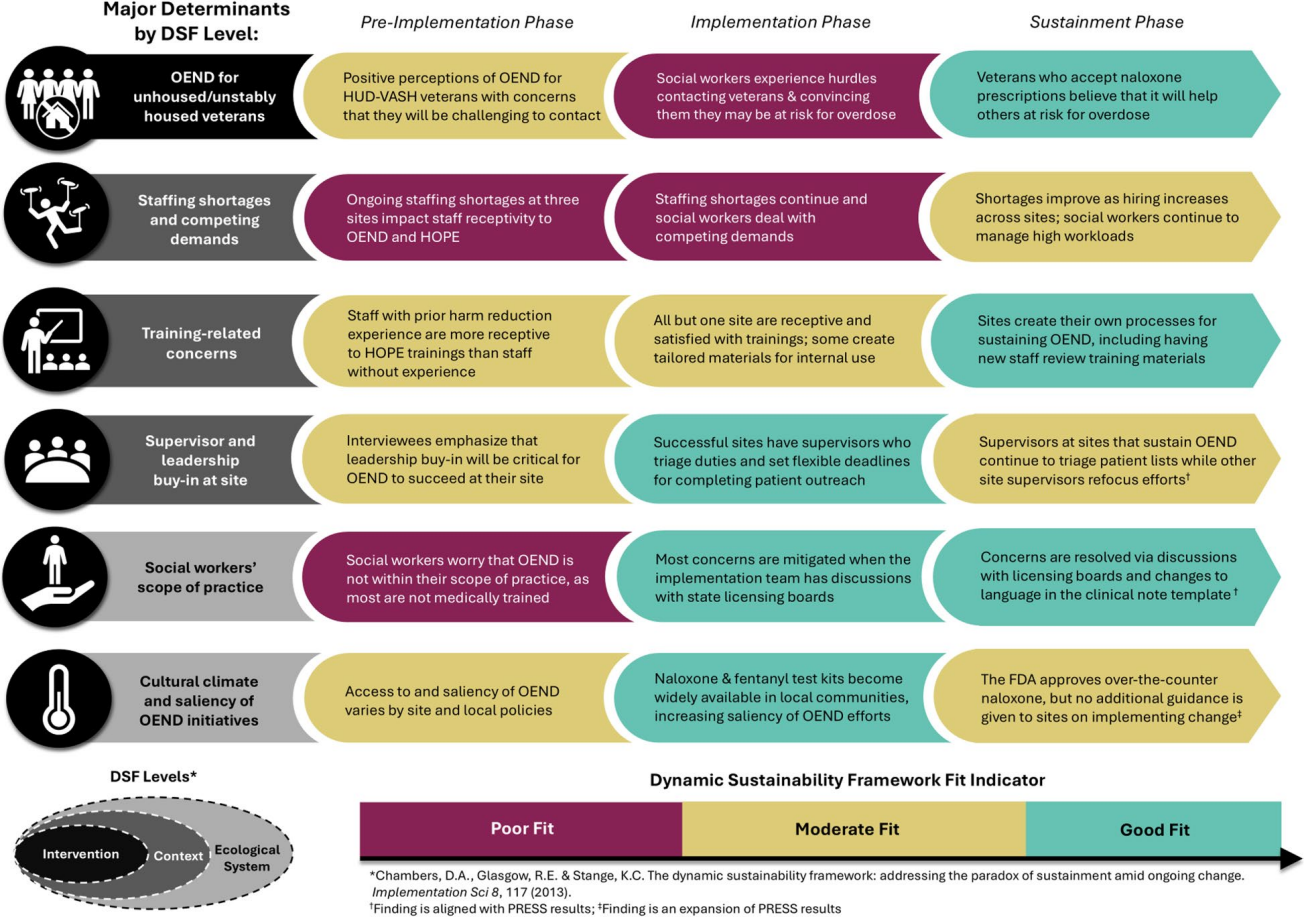
Major determinants by DSF level and fit across implementation phases

Qualitative results are organized based on the DSF level and relevant components. To illustrate these results over time, Fig. 3 is included, which provides an overview of how key determinants (i.e., impacted tailoring of the initial implementation approach) changed across the three phases. Each of these key determinants is identified with an indicator of fit (i.e., poor, moderate, or good).

### DSF level: intervention

#### Components (PI, I, S)

##### Intervention components (PI, I, S)

The intervention is provision of opioid overdose education (OE) by a social worker and naloxone distribution (ND) by a prescriber at the VA site to an at-risk HUD-VASH veteran. Once the social worker provides OE, they complete a templated clinical note in the electronic health record that alerts the prescriber whether the veteran accepts or declines a naloxone prescription. If the veteran accepts naloxone, the prescriber reviews the veteran’s chart for relevant clinical information, fills and releases naloxone by mail or for in-person pick-up, and answers any medication-related questions the veteran or social worker may have.

##### Positive perceptions of OEND for unhoused and unstably housed veterans (PI, I, S)

Interviewees across sites recognized the importance of OEND for the HUD-VASH population prior to implementation:

*I myself personally [and] most of my colleagues all believe in the access to {naloxone}.* [2-S-06, Supervisor]

The perceived value of OEND varied by site and appeared to have a connection with the saliency of overdoses in the community:

*First, recognizing the statistics on… overdoses and suicide risk or death risk in each area and realizing that this is a fairly simple process that doesn't take a lot of time…* [1-S-01, Program Manager]

##### Social workers as intervention practitioners (PI, I)

Prior to implementation, we interviewed site prescribers who would be responsible for ND. Many interviewees identified social workers as ideal candidates to complete OE since they have pre-existing relationships with HUD-VASH veterans. Yet, during implementation, some social workers felt discomfort about providing OE since they are often not medically trained:

*Case managers have such an intimate relationship. We have ongoing case management where [we’re] in the veteran's homes, we have a very unique relationship with our veterans ... we built a lot of trust over the years. So I get … why it's important … for case managers to … address this issue, but at the same time, there's a lot of case managers that are not comfortable with this program because it's a little bit more medical related.* [3-S-13, Social Worker]

##### Progress towards OEND-related outcomes (S)

During sustainment, we asked interviewees about progress toward primary OEND outcomes (i.e., increased knowledge via education about opioid overdose risk and naloxone among HUD-VASH veterans and staff, more veterans receiving naloxone prescriptions since implementation ended). Across all sites except for one, HUD-VASH staff reported that competing initiatives at the site had decreased OEND volume, but OEND remained a priority. This response was echoed by site prescribers, who reported that naloxone requests from HUD-VASH social workers had decreased since OEND implementation ended.

#### Delivery platform (PI, I, S)

##### Timing of intervention (PI)

During pre-implementation, respondents across multiple sites said that OEND should be introduced to veterans during HUD-VASH enrollment but revisited during one-on-one meetings with social workers. However, respondents at two sites preferred that social workers have OE conversations once veterans in the program are housed, which may occur months after enrollment.

Notably, across sites, social workers generally relied on veterans to self-disclose substance use, which would provide an opportunity to discuss OE. However, they emphasized that veterans may be unwilling to disclose use until the social worker has built rapport with them.

##### Mode of delivery (I, S)

During both implementation and sustainment, social workers reported completing OE via phone outreach or during face-to-face visits with veterans in an office setting. During sustainment, some social workers reported completing OE in the field during home visits with veterans. Prescribers generally did not meet with veterans face-to-face but provided outreach via phone.

##### Potential challenges (I, S)

During implementation and sustainment, interviewees reported several challenges to OEND delivery, including difficulty contacting unstably housed veterans and challenges with dispensing naloxone to veterans.

### DSF domain: Practice setting/context

#### Staffing (PI, I, S)

##### Staffing challenges (PI, I, S)

During implementation, interviewees at three sites expressed that ongoing staffing shortages were impacting staff availability. Staffing issues were reported by interviewees as mostly resolved during sustainment, as hiring increased across sites between implementation and sustainment interviews.

##### Social worker workload burden (S)

During sustainment, social workers and mid-level leaders across sites stated that social workers in HUD-VASH have a high workload burden, making it difficult to dedicate time to think about and participate in OEND implementation:

*Probably one of the challenges that we have faced in implementing OEND … it's like all the spinning plates in a circus act, … trying not to drop any plate and sometimes plates get dropped*. [2-S-03, Supervisor]

##### Other important personnel for OEND efforts (PI, S)

During pre-implementation, three sites reported that their HUD-VASH program did not currently have a designated prescriber that would work with veterans. However, interviewees agreed that having a prescriber was essential for implementation success:

*[The prescriber] is also a very good source of information… a lot of times she is able to answer a lot of questions. If people have confusion on well, which note title do I use like do I use the consultation … she's able to help refresh people's memories on that.* [4-S-02, Social Worker]

During sustainment, interviewees also identified other individuals who could assist with implementation, including program analysts, nursing staff, clinical pharmacists, substance use disorder specialists, and peer support specialists. These individuals were either peripheral to or embedded in the HUD-VASH program:

Our SUD specialist is our biggest champion of OEND. [1-S-05, Supervisor]

Most of our peer specialists are in recovery … because they're kind of in their recovery mindset … and so they're somebody who I think is probably more comfortable… having that chat with the veteran. [1-S-11, HUD-VASH Staff]

#### Information systems (PI, I, S)

##### Electronic Health Record clinical note (EHR; PI, I, S)

During implementation, interviewees at sites A, B, and D also expressed hesitancy to complete the EHR-based templated clinical note since it included language about educating the patient about medication (i.e., naloxone). One state’s social work licensing board deemed discussing medication as outside the social work scope of practice, even in the context of harm reduction. In response, the note template was converted to one that allowed the social worker to select it was outside their scope of practice, which social workers indicated they were less hesitant to use.

##### Technical assistance (PI)

During pre-implementation, interviewees across sites mentioned wanting technical assistance to help them fulfill their OEND duties. In particular, interviewees mentioned wanting tracking tools to monitor outreach, reminders/pop-ups, and training on consistent documentation.

During sustainment, two sites noted they were able to establish a workflow for pulling veteran lists from the web-based, case-finding dashboard, while other sites did not have an established workflow. At sites A and B, the HUD-VASH program manager accessed the dashboard to identify eligible veterans and distributed lists to supervisors. Supervisors then notified corresponding social workers or other HUD-VASH staff on their teams to complete assignments.

#### Organizational culture/climate structure (PI, I, S)

##### Prior knowledge and experience impacts comfort with OE (PI, I, S)

We found that prior OEND-related knowledge and experience were important components of OEND implementation across sites and across all phases. Staff with experience in substance use disorder treatment settings and with harm reduction approaches found OE especially easy, as they were able to frame OEND as a helpful tool to have on hand during emergencies.

In contrast, there were HUD-VASH social workers across all sites who were new to and expressed discomfort with OEND. These attitudes persisted at sustainment, with some interviewees noting continued hesitancy:

*I think some of our case managers still struggle with it. Some of it is just that … they're newer social workers and … they're not necessarily as comfortable in their roles yet. And, also, …substance use isn't their specialty. …I think it can be a little daunting at times.* [1-S-11, HUD-VASH Staff]

#### Training (I, S)

##### HOPE training content (I, S)

During implementation and sustainment, three sites provided positive feedback about training content and resources provided. In contrast, one site’s feedback was mixed. They noted it was helpful to have a social worker on the implementation team available during trainings to answer questions, but some social workers may never feel comfortable delivering OEND.

##### Training resources and boosters (I, S)

During implementation and sustainment, all sites had used materials from trainings to bolster OEND delivery. Three sites developed their own tailored materials from trainings. One HUD-VASH program manager said:

*I created a two-pager, a how-to checklist for staff because there was so much information in the beginning, you know the [electronic health record note] information, the prescribers, the criteria providing education….* [1-S-01, Program Manager]

Some sites also developed their own methods for distributing resources. At one site, a HUD-VASH program assistant and peer support specialists mailed educational materials to HUD-VASH veterans who expressed interest in OEND. At another site, OEND materials were stored in a centralized folder and reviewed during new employee orientation.

During sustainment, interviewees across all sites mentioned wanting a refresher or booster training for HUD-VASH staff. Sites C and D requested periodic e-mail communications from the HOPE team with reminders to complete OEND, links to new OEND resources, and updates from the field. Some interviewees also suggested new topic areas for future OEND trainings; for instance, sites A and B noted the importance of continued education about new overdose risk groups (e.g., individuals with stimulant use disorders).

##### Incorporating OEND in onboarding processes (I, S)

During implementation, HUD-VASH interviewees described incorporating OEND into new staff onboarding processes. Strategies included: 1) having new staff review OEND slides and training materials; 2) suggesting new hires complete virtual OEND trainings on VA’s training platform; 3) keeping OEND as an agenda item during regular staff meetings; and 4) continuing to emphasize the importance of OEND for HUD-VASH veterans and normalizing having these conversations with veterans.

During sustainment, interviewees at all four sites agreed that training new hires is a crucial part of OEND success in the short-term. Some sites established infrastructure to continue OEND in the long-term, with a program manager noting,*”It's part of our orientation for new social workers”* [1-S-01, Program Manager]. Another interviewee stated:

*Now that they've embedded it as part of the new employee orientation, it's just another expectation with everything else that they do.* [2-S-05, Leadership]

#### Leadership and supervision (PI, I, S)

##### Mid-level leadership buy-in (PI, I, S)

During pre-implementation, multiple interviewees across sites noted that mid-level leadership (i.e., site leadership, including Chief Medical Officers) buy-in would be an important facilitator of OEND implementation. Sites expressed that HUD-VASH mid-level leaders were responsible for setting expectations regarding OEND:

*When I brought [OEND] up to the Chief of Primary Care … you know that was her same response too … we should already be doing [OEND], … this is best practice, this should be our standard of care … I think just having that culture of, “This is just what we do.” That's how we're going to sustain them.* [2-S-05, Leadership]

Yet, during implementation, integration of OEND into everyday workflow varied by site. At site A, the HUD-VASH program manager played an active role in supporting OEND efforts by setting deadlines for staff to complete veteran outreach, working with staff to create their own tailored materials for completing OEND, and supporting individual teams in developing processes that worked for them. At site C, the HUD-VASH program manager was supportive of OEND efforts but managed several de-centralized sites; thus, they were not able to take on a more active role in supervising these efforts. At both sites B and D, HUD-VASH leadership were not prioritizing OEND during implementation due to competing demands and concerns with scope-of-practice.

During sustainment, OEND workflows were further refined at some sites and stagnated at others. At sites A and B, HUD-VASH mid-level leaders took on the responsibility of pulling OEND-eligible veteran lists from a web-based, case-finding dashboard and distributing lists to their teams:

*It all comes from me down, from leadership down. So it's going to be me being consistent and getting that list out every month and continuing that conversation. Doing the office hours, making sure it is included in orientation, and just making sure that people understand the importance of it.* [2-S-08, Program Manager]

At sites C and D, some interviewees mentioned including OEND as an agenda item in meetings but did not mention structured efforts spearheaded by leaders.

##### Supervisor engagement (I, S)

Interviewees described that integration of OEND into staff workflow varied due to supervisory differences. During implementation, HUD-VASH supervisors set the tone for social workers’ expectations regarding OEND, including at site B:

*[HUD-VASH] Staff have done the training but there was a lot involved in the trainings. With, you know, so it's like just a lot of information… and I think that there wasn't clear guidance from us as leadership on what is expected of our staff.* [2-S-02, Supervisor]

In contrast, supervisors who had established workflows for their teams (e.g., via deadlines) experienced more success with continuing OEND:

*I think the communication between supervisor and whoever is to make the calls to reach out to the veterans is good and then there are deadlines that we get … the deadlines help, because then that provides some urgency.* [1-S-18, Social Worker]

During sustainment, many supervisors relied on the HUD-VASH program manager to send lists of eligible veterans from the web-based, case-finding dashboard, yet reported not having received these lists since implementation ended. Supervisors and social workers also mentioned prioritizing other issues over providing OEND during the sustainment period.

### DSF domain: ecological systems

#### OEND in other practice settings (PI, I, S)

Some interviewees mentioned that some VA personnel were already involved in sitewide OEND efforts spearheaded by pharmacy, behavioral health, substance use programs, and VA police. Across phases, interviewees perceived that OEND could be sustained in HUD-VASH by involving staff from these other, sitewide efforts.

Across all sites, interviewees noted that opioid overdose risk awareness in local communities enhanced OEND implementation and sustainment, with many veterans and staff alike aware of naloxone and its importance:

*The community’s fairly aware I think of … fentanyl and … that being a risk factor in the community. And so I think that that’s sort of actually been a help because people are like oh yeah, that’s something like we need to worry about. That’s something that … we have to pay attention to. And so I think that’s actually … helped me because I’ve even had veterans be like, … even if I don’t use it for me …it’s good to have it.* [1-S-11, HUD-VASH Staff]

Yet, many interviewees across all sites were unfamiliar with non-VA community resources during pre-implementation interviews. Three HUD-VASH programs did not have any formal community partnerships at this time-point; however, some individual social workers had informal community contacts. During sustainment, two sites specified that there were community resources available that could aid in their OEND efforts. Interviewees at these sites also mentioned that they had non-VA social worker colleagues who can carry and administer naloxone in the field. As one interviewee noted:

*The more steps, the more difficult it is to have veterans get naloxone. They shouldn’t be required to have to go to the pharmacy. Often, veterans [needs] are being met within the community.* [3-S-16, Social Worker]

#### Policy (PI, I, S)

##### HUD-VASH expansion policy (PI, I, S)

During all three timepoints, we asked about the HUD-VASH expansion – the Veteran HOUSE Act of 2020—to increase access to resources for veterans who were previously ineligible (e.g., Other Than Honorable discharge status). Although this Act resulted in expansion of eligibility for certain HUD-VASH services for a larger number of veterans, there was uncertainty about whether these newly eligible veterans were eligible to receive OEND from the VA [24]. Some interviewees were uncertain about how this policy would affect their veterans, with some interviewees mentioning that they connect these veterans to medical care in the community or via Medicaid. During sustainment, Site B noted that these veterans seem to make up a large portion of their veteran population, but social workers at two other sites noted said they had not yet encountered any veterans who were affected by the policy.

#### Regulations (PI, I, S)

##### Social workers’ scope of practice (PI, I,)

Sites in one state had concerns that OEND was outside of social workers’ scope of practice and was not aligned with state licensing laws for social workers. Concerns were ameliorated via conversations between the implementation team, state licensing board, and high-level leaders at these sites during implementation. At sustainment, these concerns were resolved.

##### Social workers carrying and distributing naloxone (S)

During sustainment, social workers at three sites expressed that social workers should be able to carry and distribute naloxone, which was outside the focus of the HOPE trial. For instance, one social worker at Site C said:

*[When] educating [veterans] and educating ourselves quite frankly, my thought was why aren't they offering this for staff, like why don't we have this in our vehicles or on us because we're going into these homes and you know what I mean?* [3-S-19, Social Worker]

#### Market forces (PI, I, S)

##### Local and state naloxone distribution (PI, I, S)

Prior to OEND implementation, naloxone access outside the VA varied by state. During the implementation period, several interviewees mentioned that naloxone and fentanyl test kits were becoming more widely available within the non-VA community:

*In [geographic area], there seems to be a lot more, and I’m not even talking about VA, just community providers being able to have [naloxone]. So it’s just this culturally more accepted practice. Umm. And it would be great to have that sort of spread throughout our cache, throughout our Medical Center, but I’m not sure because we are a Medical Center and hierarchy that way that it will ever change, which is fine.* [3-S-02, Leadership]

##### Over-the-counter distribution of naloxone (S)

During sustainment interviews at Sites A and B, over-the-counter distribution of naloxone was approved by the Food and Drug Administration. However, none of our sites had yet received instructions from VA on how to engage with this change. One prescriber indicated that this approval could impact other populations at risk for overdose.

#### Population characteristics (PI, S)

##### Unhoused and unstably housed veterans (PI, S)

During pre-implementation, interviewees at all sites described HUD-VASH veterans as being hard-to-reach, which would be a barrier to OEND outreach. At this time-point, there was also a common concern among interviewees that veterans would not want to discuss substance use until rapport had been built with their assigned social worker. During sustainment, the most common reason for veterans declining OEND across all sites was that veterans did not believe they were at risk for overdose for various reasons (e.g., they do not use substances currently). Yet, we did find that some HUD-VASH veterans accepted a naloxone prescription to help others at-risk:

*I’ve had vets tell me that they didn’t think they needed it because, you know, they were prescribed opioids, and they always take it properly. But I’ve had some tell me, well, you know, I just saw a guy in the elevator in my building the other day. … we had to call 911 for him and they would have been handy to have something like that. So yeah, I’ll take it because I think there’s people in my building that need it.* [1-S-05, Supervisor]

##### Prevalence of stimulant use disorders in HUD-VASH population (PI)

During pre-implementation interviews, we asked interviewees if opioid use disorders or stimulant use disorders were more prevalent among their veteran population. Interestingly, many interviewees identified stimulant use disorders as a more common issue for veterans across all four sites.

### PRESS results and synthesis

All individuals who completed interviews during the sustainment period also completed the PRESS. Table 5 presents a summary of PRESS results by site and respondent type.

**Table 5:** Results from the provider report of sustainment scale by site and respondent type

	PRESS Item					
	1. Staff use OEND as much as possible when appropriate		2. Staff continue to use OEND throughout changing circumstances		3. OEND is a routine part of our practice	
	M	SD	M	SD	M	SD
All Respondents (N = 23)	2.11	1.00	2.15	0.97	2.78	1.27
By Site						
Site A (*n* = 6)	2.17	0.75	2.33	0.82	3.42	0.49
Site B (*n* = 4)	2.50	0.58	2.75	0.96	3.00	1.15
Site C (*n* = 7)	1.57	1.27	1.43	1.13	1.71	1.50
Site D (*n* = 6)	2.42	1.02	2.42	0.49	3.25	0.99
By Respondent Type						
Program Manager (*n* = 3)	2.00	1.00	2.33	0.58	3.17	0.29
Supervisor (*n* = 5)	1.60	1.14	1.60	1.14	1.40	1.34
Program Coordinator (*n* = 2)	2.25	0.35	2.25	0.35	1.75	0.35
Social worker (*n* = 4)	2.00	1.41	2.00	1.41	2.50	1.29
Other Personnel (*n* = 2)	2.50	0.71	3.00	0.00	3.50	0.71
Prescriber (*n* = 7)	2.43	0.98	2.29	0.95	3.86	0.38

On average, respondents reported that staff use OEND as much as possible when appropriate (Item 1) and staff continue to use OEND throughout changing circumstances (Item 2) to a moderate extent. Of note, Site D’s responses skewed higher on both items when compared to other sites. These findings align with qualitative results: At sustainment, as Site D reported higher engagement with OEND due to changes to the EHR clinical note template language and resolved concerns related to scope of practice.

When asked if OEND is a routine part of practice, responses varied. Site C considered OEND a routine part of their practice “to a slight/moderate extent” (M = 1.43). In contrast, Site A considered OEND a routine part of their practice “to a great/very great extent” (M = 3.42). These findings are also supported by qualitative results: while some sites chose to refocus efforts at sustainment (e.g., Site C), others continued their efforts (e.g., Site A).

When examining trends across respondent type, HUD-VASH Supervisors’ responses indicated slightly less use of OEND during sustainment, with average responses ranging from M = 1.40 on Item 3 to M = 1.60 on Items 1 and 2. In contrast, Prescribers and Other Personnel’s responses indicated higher OEND use at sustainment, ranging from M = 2.50 on Item 1 to M = 3.50 on Item 3 for Other Personnel and M = 2.29 on Item 2 to M = 3.86 for Prescribers. We found that these results were partially explained in our qualitative analyses: supervisors at some sites reported pivoting to other priorities during sustainment, while prescribers persisted with OEND efforts.

## Discussion

This longitudinal, multi-methods evaluation identified six key determinants that influenced the reciprocal fit of the intervention, practice setting, and ecological system: (1) OEND for unstably-housed veterans; (2) staffing shortages and competing demands; (3) training-related concerns; (4) supervisor and leadership buy-in at site; (5) social workers’ scope of practice; and (6) cultural climate and saliency of OEND initiatives. While the core components of OE and ND did not change over the course of the study, their fit changed over time. In this section, details regarding the optimization of fit are provided, followed by lessons learned for future implementation studies.

The HOPE trial was designed to deploy the implementation strategies of educational outreach, audit and feedback, and creation of novel clinical teams [25]. Regular educational outreach helped to address the first determinant—OEND for unstably housed veterans. Findings from the pre-implementation phase helped to refine the educational approach at the first two implementation sites. For example, findings indicated that it was critical to tailor the introduction to and framing of the need for OEND based on the individual veteran’s history and needs, including addressing stigma, an important barrier to harm reduction in this population [26]. As such, training for social workers introduced several messaging approaches, including focusing on the benefits of having naloxone on hand to help others and framing naloxone as analogous to a fire extinguisher (i.e., have it in case of an emergency). Interviews during the sustainment phase revealed that HUD-VASH staff had success with being flexible in their framing of OEND.

Staffing shortages and competing demands are a consistent challenge in implementation, and team-based approaches can be critical to address this determinant [26]. There are documented shortages in the number of social workers hired throughout the United States relative to the total number of people who need these services [27]. Unsurprisingly, the pre-implementation evaluation revealed that this determinant needed to be addressed in this trial. Consequently, the implementation team created novel clinical teams, which included the addition of at least one prescriber who was not part of the HUD-VASH care setting. As revealed in implementation and sustainment evaluations, this novel clinical team did not ameliorate all staffing shortages. However, their continued presence during the sustainment phase allowed OEND to continue in the HUD-VASH setting, even though it was not always an optimal fit.

Related to competing demands, at pre-implementation, site-level staff were concerned with finding time to complete trainings and adding OEND on top of social workers’ existing responsibilities, especially for those who were not already doing harm reduction. Thus, educational outreach was streamlined and materials were designed to be user-friendly and used for reference when doing OEND with a veteran. Evaluations at subsequent stages revealed that teams at the different sites continued to rely on these user-friendly training materials to make their own tailored reference guides for completing OEND. Some sites started using materials regularly for new staff onboarding.

Site-level supervisors were critical to ensuring that tailored guides were developed to meet the needs of their staff, with both supervisors and regional leadership contributing to enhanced fit of OEND in HUD-VASH. Specifically, leaders identified both educational outreach and OEND with at-risk veterans as important priorities for HUD-VASH staff. The sustainment evaluation, including PRESS findings, revealed that supervisors at sites worked to continue OEND by providing additional staff support when possible, such as analyst time to provide social workers with lists of patients on their panels eligible for OEND. Given competing demands, including ensuring housing for veterans, some supervisors had to sometimes refocus efforts, which meant OEND remained only a moderate fit at their sites.

Supervisors at sites were also key to alerting the implementation team to HUD-VASH social workers’ concerns about OEND being outside their scope of practice. At one site, monthly audit and feedback reports revealed early on that a site was not yet providing OEND, which in turn prompted a conversation between the supervisor and HOPE implementation team. It was during this conversation that concerns with scope of practice were revealed. This necessitated the implementation team to initiate a meeting with a state board of social work to determine the validity of these concerns and how they may impact OEND. Through these meetings, we clarified that overdose education was within the social work scope of practice, and a change in workflow ameliorated concerns and allowed the trial to progress. These actions also eventually led to a change in the EHR-based clinical note to enable social workers to indicate if they felt OEND was outside their scope of practice while still alerting the prescriber to complete the OEND. Follow-up qualitative and quantitative (PRESS) findings revealed that these sites achieved a good fit of OEND during sustainment. This determinant also highlights the importance of tailoring implementation approaches as needed, which is a growing area in implementation science, including evaluation of more systematic methods for tailoring [28].

The final key determinant identified – cultural climate and salience of OEND initiatives – was one that evolved across the course of the implementation trial and continues to evolve to this day. At the initiation of the trial in 2021, naloxone was only available with a prescription [29], then became available over-the-counter in 2023 [30]. However, over-the-counter availability has not extended to VA. Current guidelines state that veterans are required to see a VA provider to obtain a free naloxone prescription, and any over-the-counter naloxone would need to be purchased [31]. Furthermore, during this trial some VA sites began implementation of fentanyl test kits as a promising harm reduction strategy, including as part of larger harm reduction programs [32]. Although evidence for their use in reducing overdose death is not clearly established, recent research indicates their promise in reducing overdose risk [33]. These changes, and others occurring at both the national and local level, may present future challenges in determining OEND fit in the VA HUD-VASH setting. However, findings from the sustainment phase indicate HUD-VASH staff continue to see OEND as at least a moderate fit. It may be the case that more community-based partnerships with local harm reduction entities will be needed to ensure unstably-housed veterans at risk for overdose receive necessary harm reduction services.

### Lessons learned

While the initial pre-implementation evaluation was useful for refining the implementation approach, identifying scope of practice as a determinant emphasized the need to be flexible, responsive, and modify our approach as needed. One lesson learned was the importance of using existing implementation science tools to document modifications to the implementation approach (e.g., the Framework for Reporting Adaptations and Modifications to Evidence-based Implementation Strategies) [34], thus enabling further evaluations of mechanisms of change. Fortunately, the DSF-guided longitudinal evaluation helped us to evaluate the reciprocal fit of the intervention in the given setting from pre-implementation to sustainment. However, the supplemental use of the revised CFIR was helpful to ensure a common set of definitions when applying the DSF and assessing fit throughout our trial. Importantly, adding a simple implementation measure (i.e., PRESS) at sustainment helped provide additional validation of qualitative data.

### Limitations

First, the methods used in this study required adequate staffing and funding over time, which may not be possible for all teams embarking on this work. Many teams cite lack of funding and resources as a barrier to measuring sustainment of evidence-based interventions [35]. Second, the study was conducted in the Western region of the United States and findings may not be generalizable to other regions and may have limited generalizability to non-VA settings serving unstably housed individuals.

## Conclusions

This study addresses a gap in the literature, as many implementation studies do not systematically document methods and measures from pre-implementation through sustainment [35]. It demonstrates the value in applying theoretical frameworks systematically over time, including for evaluating the overall fit of the intervention in the given setting and context. Findings are in alignment with the DSF that “an intervention should not be optimized prior to implementation, or even prior to ‘sustainability phase’ onset.”[19] Long-term sustainment of OEND ultimately depends on the capacity of leadership and practitioners at sites to adapt processes to meet their needs throughout changing circumstances. To achieve the goals set forth in the 2022 White House Drug Control Strategy and reduce overdose deaths [6], it is imperative for both implementation scientists and practitioners to document the methods and measurements used to implement and sustain OEND interventions across diverse settings and populations. The methods presented in this study represent a rigorous approach for capturing key determinants of implementation and sustainment of OEND in a high-risk setting, which others can build upon.

## Supplementary Information

Supplementary Material 1.

Supplementary Material 2.

Supplementary Material 3.

Supplementary Material 4.

## Data Availability

The dataset analyzed for the current study are not publicly available as they contain personally identifiable information. Redacted data are available from the corresponding author on reasonable request.

## Abbreviations

CFIR 2.0: Consolidated Framework for Implementation Research (2.0)
DSF: Dynamic Sustainability Framework
EHR: Electronic Health Record
HOPE: Homeless Overdose Prevention Expansion
HUD-VASH: U.S. Department of Housing and Urban Development – VA Supportive Housing
I: Implementation
OEND: Opioid Overdose Education and Naloxone Distribution
PBM-ADS: Pharmacy Benefits ManagementAcademic Detailing Services
PI: Pre-Implementation
PRESS: Provider Report of Sustainment Scale
S: Sustainment
VA/VHA: Veterans Health Administration
VAHCS: Veterans Affairs Healthcare System
VISN: VA Integrated Service Network
