# Supplementary material for: Longitudinal evaluation examining implementation and sustainment of an opioid overdose education and naloxone distribution among veterans who are unstably housed
Source: Implement Sci Commun. 2025 Aug 6;6:83. doi: 10.1186/s43058-025-00764-3 (PMC12330058; doi:10.1186/s43058-025-00764-3)
Supplement: Supplementary file 1 — Supplementary Material 1. [file 43058_2025_764_MOESM1_ESM.docx]

**Appendix A. Pre-Implementation Interview Guide**

The objective of this quality improvement initiative is to prevent opioid-related overdoses by increasing Veterans’ access to opioid overdose education and naloxone (OEND). Specifically, we want to make sure Veterans residing in HUD-VASH who have an opioid use disorder or who are taking opioids, receive annual OEND. This interview will focus on identifying factors that may impact implementation of OEND at your site. Are you familiar with OEND?

*[If they say no, then tell them a bit about it]* [Short blurb about OEND] The VA OEND Program aims to reduce harm and risk of life-threatening opioid-related overdose and deaths among Veterans ([LINK](https://www.pbm.va.gov/PBM/academicdetailingservice/Opioid_Overdose_Education_and_Naloxone_Distribution.asp)).

- Two main components:
  - Education and training regarding opioid overdose prevention, recognizing sign of overdose, and overdose rescue response
    - HUD-VASH staff such as social workers will help with educating Veterans
  - Distribution of naloxone/Narcan (nasal spray)
    - The HOPE team is working on identifying someone at your site to prescribe the Narcan

Are you familiar with naloxone? *[If they say no, then provide brief description]* Naloxone, commonly known as Narcan, is a medicine that rapidly reverses an opioid overdose and is usually given as a nasal spray or injection.

Background Information about Interviewee:

- Name
- Role within VA/organization, length of time in role, length of time in VA/organization
- Educational background/Professional training
- Other

Contextual Factors:

Before we dive into questions about OEND, I want to first get a general sense of your HUD-VASH site.

- Could you give me an estimate of the current patient panel size at your HUD-VASH site? What is your average case load?
- How many Veterans in your case load or patient panel…
  - Have a dx of OUD?
  - Are on long-term opioid therapy (LTOT)?
  - Have a diagnosis of stimulant use disorder?
  - [Maybe also] Are at risk for overdose?
- Do you have any academic detailers at your HUD-VASH site?
  - *[If yes]* What are their roles?
- Do you have prescribers (e.g., MDs) at your HUD-VASH site who you are working with? What are their roles?
  - [Example prompts if interviewee is a prescriber] Do you work with other prescribers at your site? What is the process for Narcan prescribing if you are away from work (e.g. out of office)?
- Have you or your HUD-VASH site had any previously experience with implementation or quality improvement efforts?
  - *[If yes]* Could you describe these efforts?

Current State:

First, I would like to discuss how things currently work within HUD-VASH at your site.

- What is your current process for identifying patients who may be at risk for overdose?
  - *[Probe]* Do you verify this through medical records?
- How is your HUD-VASH program organized? Do social workers have generalized or compartmentalized roles?
  - *[Probe]* Intake, Housing, Long-term case management
- How does your HUD-VASH program currently help Veterans who are at risk of overdosing on opioids?
- How does your HUD-VASH program currently help Veterans who may have stimulant use disorders?
- Do you think HUD-VASH should do more to decrease rates of overdose among homeless Veterans, and if so, what? If not, what do you think it is doing well and why?
- Are there agencies other than the VA that help reduce rates of overdose or provide Narcan among homeless or unstably housed individuals? How does this process work?
- Have you observed any of your clients experience a reversal, and if so, how did this impact your views on using Narcan to prevent overdoses?

Input on Intervention Approach and Components:

I’d like to now talk about ways to improve OEND at your site.

- What are your initial perceptions of the intervention and perceived value to Veterans in HUD-VASH?
- [Prescriber only] Have you ever prescribed Narcan to a Veteran?
  - If yes
    - Under what circumstances did you prescribe Narcan (probes: Veteran asked for it; clinical indication for opioid use disorder in CPRS; other)?
    - What’s your comfort level delivering OEND?
  - If no
    - Would you prescribe Narcan? Under what circumstances (probes: Veteran asks for it; clinical indication for opioid use disorder in CPRS; other)?
- What changes need to be made for OEND/Narcan distribution [gauge interviewee’s preferred term] to work within your HUD-VASH program?
- What might get in the way of providing OEND to Veterans in in HUD-VASH?
  - What additional resources are needed to address these barriers?
- Which staff members will need to be involved in improving OEND and why?
- When do you think would be the best time to target Veterans in HUD-VASH with this intervention, and why?
  - *[Probe]* Entry into HUD-VASH, or other criteria? E.g., intake, apt move-in?
- Have you ever seen a Narcan order documented in a medical record?

Outcomes and Evaluation:

- What do you hope will change from greater use of OEND?

Implementation Strategies:

- What kind of support would be helpful to ensure implementation of OEND at your site?
  - *[Probe]* How could we make this easier for you? Do you know who to target?
- Who should be included in trainings? Other outreach/education activities?
- What do you think are the best strategies for communication about implementation of the intervention, including initialization, barriers, and successes?
- Any strategies come to mind that have worked in the past when you implemented new programs/services?

Ecological System:

- [Leaderships only – BONUS] HUD-VASH Expansion Policy: Recently, Congress passed legislation to expand eligibility for the HUD-VASH program to include homeless Veterans whose military discharge status (i.e., “Other than Honorable” status) made them ineligible for housing benefits. Source: ([LINK](https://endhomelessness.org/positive-changes-in-fy2021-spending-hud-vash-eligibility-expands/))
  - Have you heard about this policy?
  - What are your perceptions of this policy expansion?
  - How has this policy affected implementation of OEND at your HUD-VASH program? Alternately, how do you think this policy may affect OEND implementation at your HUD-VASH program?
- Are there any other local/state/federal policies that may affect the implementation of OEND at your site? For example, state and local laws about carrying Narcan?
- Are there any agencies/resources available outside the VA to assist in access to OEND?

Sustainability: *[this would be the first to cut if running out of time]*

- How does your HUD-VASH program keep in contact with HUD-VASH graduates?
- Who are the people at the site that would be involved in making decisions about sustaining the intervention?
- What kind of information/evidence is needed to influence decisions about sustainability? (e.g., may be useful to probe for information about clinic codes, VERA reimbursement considerations, performance metrics of interest, etc.)

Wrap-up:

- Is there anything else you’d like to share with me?
- Who else at your site do you think would be essential for us to interview?

Parking Lot:

- Can you walk me through the process of how Veterans gain entry into HUD-VASH?
