# Supplementary material for: Longitudinal evaluation examining implementation and sustainment of an opioid overdose education and naloxone distribution among veterans who are unstably housed
Source: Implement Sci Commun. 2025 Aug 6;6:83. doi: 10.1186/s43058-025-00764-3 (PMC12330058; doi:10.1186/s43058-025-00764-3)
Supplement: Supplementary file 2 — Supplementary Material 2. [file 43058_2025_764_MOESM2_ESM.docx]

**Appendix B. Implementation Interview Guide**

Hello, *[participant].* Thanks for taking the time to meet with me today.

The purpose of today’s interview is to learn from you about how OEND implementation is going at your HUD-VASH site now that our OEND educational outreach training is underway.

Just as a reminder, the VA OEND Program aims to reduce harm and risk of life-threatening opioid-related overdose and deaths among Veterans.

- Two main components:
  - Education and training regarding opioid overdose prevention, recognizing sign of overdose, and overdose rescue response
    - HUD-VASH staff such as social workers will help with educating Veterans
  - Distribution of naloxone/Narcan (nasal spray)
    - The HOPE team has identified a prescriber at your site to help distribute Narcan.

**Background Information about Interviewee:**

- Name
- Role within VA/organization, length of time in role, length of time in VA/organization
- Educational background/Professional training
- Other

**Implementation:**

- *[For sites with good implementation progress]* We are thrilled with [site’s] progress. In your opinion, what do you think has made OEND implementation go so well?
- *[For sites w/ minimal progress]* I understand that OEND implementation at [site] has been challenging. Can you tell me about what has been challenging?
  - How have you or other HUD-VASH staff tried to address these challenges?
- *[Prompt for both types of sites]* When thinking back over these past couple of months, what would you say are the top 2-3 barriers your site has faced with implementing OEND?
  - *[Probe if they just name the barriers]* Can you tell me more about these barriers?
- We also want to know what has worked well for your site. Can you tell me more about what has been successful at your site?
- How easy have you found it for staff to integrate OEND into their workflow?
- We know from our performance feedback that some Veterans are going to refuse OEND if offered it. Any thoughts or strategies that you or the staff have on how to address Veterans that refuse OEND?
- How do you think we could improve our trainings?
  - How could we improve our performance feedback? *[May need to prompt about slides shown during training on how well the site is doing]*

**Maintenance:**

- As you are aware, our trainings will eventually end. In your opinion, do you think OEND is something your staff will continue once trainings have ended?
  - *[probe]* Are there any key personnel or program offices that could support continuation of OEND?
- How well do you think new staff who weren’t able to participate in HOPE will be able to integrate OEND in their workflow?
  - What types of resources, either at the site or otherwise, do you think new staff would need to be able to integrate OEND into their workflow? *[Prompt: TMS trainings, etc.]*
- What else would you change to make OEND work at your site in the future?

**Wrap-up:**

- Is there anything else you’d like to share with me?
