# Supplementary material for: Longitudinal evaluation examining implementation and sustainment of an opioid overdose education and naloxone distribution among veterans who are unstably housed
Source: Implement Sci Commun. 2025 Aug 6;6:83. doi: 10.1186/s43058-025-00764-3 (PMC12330058; doi:10.1186/s43058-025-00764-3)
Supplement: Supplementary file 3 — Supplementary Material 3. [file 43058_2025_764_MOESM3_ESM.docx]

**Appendix C. Sustainment Interview Guide**

**INTRODUCTION**

Hello, *[participant].* Thanks for taking the time to meet with us today. I’m *[name]* and *[name]* is also joining us to take notes.

The purpose of today’s interview is to learn from you about how you plan on sustaining OEND efforts at your HUD-VASH site now that the HOPE project has finished.

Just as a reminder, the VA OEND Program aims to reduce harm and risk of life-threatening opioid-related overdose and deaths among Veterans.

- Two main components:
  - Education and training regarding opioid overdose prevention, recognizing sign of overdose, and overdose rescue response
    - HUD-VASH staff such as social workers will help with educating Veterans
  - Distribution of naloxone/Narcan (nasal spray)
    - The HOPE team identified a prescriber at your site to help distribute Narcan

We would like to audio- and video-record our conversation. Recordings will be saved on a secured drive that only the evaluation team will have access to. You can decide to have the recording turned off at any time during the interview. If you aren’t comfortable with being recorded, our team will take detailed notes instead.

Do you have any questions before we begin?

Do you agree to have our conversation recorded?

**🡪 START RECORDING**

***Questions that are bolded should be prioritized**

**I. Background Information about Interviewee:**

- Name
- Role within VA/organization, length of time in role, length of time in VA/organization
- Educational background/Professional training
- Other

**II. Sustainment Questions:**

1. ***[General question for all interviewees]*** **How have OEND efforts been going at your site?**

1. ***[For HUD-VASH staff]* How is your group *[HUD-VASH]* doing with providing OEND to eligible Veterans at your site since we last spoke on *[MONTH/YEAR WE STOPPED HOPE TRAINING AT RESPECTIVE SITE]*?**
   - **How has OEND impacted the morale of people working at your site?**
   - **How has OEND impacted workflow at your site?**

***[For prescribers]* How have OEND referrals/workflow been going at your site since we ended training [month/year]?**

- - **Any increase, decrease, or plateau in naloxone prescribing related to HUD-VASH since *[INSERT MONTH/YEAR WE STOPPED HOPE TRAINING AT RESPECTIVE SITE]?***

1. **What is the current process of delivering OEND at your site?**

*Probes:*

- - *[if participant indicates that they are delivering OEND differently than how they did during HOPE, e.g. using a different note template, not using case managers to provide education and/or harm reduction, etc.]* I see that X has changed. Could you describe to me why this change was needed?
  - *[if time allows]* Which note template are you using on CPRS for OEND referral? Could you show me the note template you are using?

1. Have there been any local policy or procedural changes that have impacted continuing OEND at your site?
   - If yes, could you describe these changes?
     - How have these changes impacted sustainability of OEND at your site?
2. **As you know, any time we create a change in clinical practice, there are a whole host of factors that could either help or hinder that change.**

**What are some factors at your VA that have helped or hindered providing OEND to Veterans at your site? Could you describe these to me?**

*Possible probes/examples you can give the interviewee if they are not sure what you mean:*

- - Characteristics of people managing/leading/supporting OEND
  - Organizational context:
    - Staffing
    - Infrastructure
    - Organizational culture
    - Management support
    - Incentives
    - Organizational mandates

1. **What are some factors outside of VA that have influenced your experience with providing OEND to Veterans at your site? Could you describe these to me?**

*Possible probes/examples:*

- Broader context
  - - Community resources
    - Local, state, or national policies
    - Regulations
    - Legal, political, or economic context
    - Patient needs/preferences, or characteristics

1. **How have Veterans responded to OEND efforts at your site?**
   - **For Veterans that declined OEND, what reasons did they give for declining?**
2. **How are you thinking OEND efforts at your site can be continued?***Probes*
   - **How are you currently or planning to on-board new social workers with OEND efforts?**
   - How are you planning on collaborating with other OEND efforts at your site?
   - How are you planning on collaborating with other homeless programs at your site (e.g., GPD, HCHV, etc.)?
   - How are you planning on collaborating with other OEND efforts outside of your site (e.g., in the community)?
3. **For current OEND efforts to continue in the long-term, what do you think needs to happen?**

*Probes:*

- - **What barriers would need to be removed?**
  - **Would staff attitudes towards OEND need to change? If so, how?**
  - Would staff incentives need to change? If so, how?
  - **What are some organizational policies that may need to change?**

1. What resources would you access for additional help with continuing OEND efforts?

*Probes:*

- - Are there any key personnel or program offices that could support continuation of OEND?
  - Who might you consider going to for additional help with continuing OEND efforts?
  - Are there any financial or tangible resources you can think of that would help OEND continue long-term?

**III. Provider Report of Sustainment Scale (PRESS) and Wrap-Up**

Before we end, I’d like to ask you three brief questions about Opioid Overdose Education and Naloxone Distribution (OEND) at your site.

Please indicate the extent to which you agree with the following items where 0-Not at all, 1-to a slight extent, 2-to a moderate extent, 3-to a great extent, and 4-to a very great extent.

1. On a scale from 0-4, how much do you agree that staff use OEND as much as possible when appropriate?
   1. Could you describe briefly why you gave this rating?
2. On a scale from 0-4, how much do you agree that staff continue to use OEND throughout changing circumstances?
   1. Could you describe briefly why you gave this rating?
3. On a scale from 0-4, how much do you agree that OEND is a routine part of your practice?
   1. Could you describe briefly why you gave this rating?

Thank you for answering these questions.

Is there anything else you’d like to share with me?

**THANK YOU!**

**🡪 STOP RECORDING**
