# Supplementary material for: Longitudinal evaluation examining implementation and sustainment of an opioid overdose education and naloxone distribution among veterans who are unstably housed
Source: Implement Sci Commun. 2025 Aug 6;6:83. doi: 10.1186/s43058-025-00764-3 (PMC12330058; doi:10.1186/s43058-025-00764-3)
Supplement: Supplementary file 4 — Supplementary Material 4. [file 43058_2025_764_MOESM4_ESM.docx]

| **Appendix D. Consolidated criteria for reporting qualitative research (COREQ)** | | | |
| --- | --- | --- | --- |
| **Domain 1: Research team and reflexivity** | | | |
| Personal Characteristics | | | |
| 1. | Interviewer | Which author/s conducted the interview or focus group? | AK, HC, MG, SJ, TP |
| 2. | Credentials | What were the researcher's credentials? *E.g. PhD, MD* | AK has an MA. HC and TP have an MS. MG has an MPH. |
| 3. | Occupation | What was their occupation at the time of the study? | All members of the qualitative team were health science specialists at the time of their participation in the study. |
| 4. | Gender | Was the researcher male or female? | All researchers identify as women. |
| 5. | Experience and training | What experience or training did the researcher have? | All researchers have training in qualitative research. SJ has completed coursework in qualitative methods. AK, HC, SJ, and TP have published studies that utilized qualitative methods. |
| Relationship with participants | | |  |
| 6. | Relationship established | Was a relationship established prior to study commencement? | Relationships were not established prior to study commencement. |
| 7. | Participant knowledge of the interviewer | What did the participants know about the researcher? | Participants were introduced to researchers in their capacity as HOPE team members but did not know their personal goals. |
| 8. | Interviewer characteristics | What characteristics were reported about the interviewer/facilitator? | Researchers were all qualitatively trained (Qualitative Methods > Data collection, p. 7). |
| **Domain 2: study design** | | | |
| Theoretical framework | | | |
| 9. | Methodological orientation and Theory | What methodological orientation was stated to underpin the study? | Directed content analysis based on the Dynamic Sustainability Framework and Consolidated Framework for Implementation Research 2.0 (Qualitative Methods > Analytic approach, p. 8). |
| Participant selection | | | |
| 10. | Sampling | How were participants selected? | Participants were purposively and selectively sampled across all implementation phases (Qualitative Methods > Data collection, p. 7) |
| 11. | Method of approach | How were participants approached? | Participants were recruited via encrypted e-mail (Qualitative Methods > Data collection, p. 7) |
| 12. | Sample size | How many participants were in the study? | 67 unique individuals participated in the study (Results, p. 8) |
| 13. | Non-participation | How many people refused to participate or dropped out? Reasons? | 64 individuals were non-responsive or lost to follow up over the course of the study. Reasons for non-participation varied, but included lack of interest or competing priorities. |
| Setting | | | |
| 14. | Setting of data collection | Where was the data collected? | Data was collected on Microsoft Teams, a secure VA videoconferencing software (Qualitative Methods > Data collection, p.7). |
| 15. | Presence of non-participants | Was anyone else present besides the participants and researchers? | No. |
| 16. | Description of sample | What are the important characteristics of the sample? | Characteristics of interviewees can be seen in Table 3 and Table 4 (p. 9). |
| Data collection | | | |
| 17. | Interview guide | Were questions, prompts, guides provided by the authors? Was it pilot tested? | Yes, interview guides for each phase are included in Appendices A-C. We did pilot test interview guides with KB, who is a social worker with experience in both OEND and VA homelessness programs (Qualitative Methods > Interview guides, p. 7). |
| 18. | Repeat interviews | Were repeat interviews carried out? If yes, how many? | Yes, repeat interviews were carried out for 18 individuals. |
| 19. | Audio/visual recording | Did the research use audio or visual recording to collect the data? | Yes, interviews were audio- or video-recorded with participants’ verbal consent (Qualitative Methods > Data collection, p. 7). |
| 20. | Field notes | Were field notes made during and/or after the interview or focus group? | Yes, interview notes were taken for each interview. |
| 21. | Duration | What was the duration of the interviews or focus group? | Interviews lasted from 15 minutes to 1 hour each (Qualitative Methods > Data collection, p. 7). |
| 22. | Data saturation | Was data saturation discussed? | No, data saturation was not discussed but data quality checks were made throughout the analytic process (Qualitative Methods > Data collection, p. 7). |
| 23. | Transcripts returned | Were transcripts returned to participants for comment and/or correction? | No, transcripts were not returned to participants. |
| **Domain 3: analysis and findings** | | | |
| Data analysis | | | |
| 24. | Number of data coders | How many data coders coded the data? | Five individuals coded interviews (AK, HC, MG, SJ, TP) (Qualitative Methods > Data collection, p. 7). |
| 25. | Description of the coding tree | Did authors provide a description of the coding tree? | We did not complete a coding tree but had a codebook based on the Dynamic Sustainability Framework. |
| 26. | Derivation of themes | Were themes identified in advance or derived from the data? | Themes were derived both inductively and deductively, as this was a directed content analysis. We began with deductive themes using constructs from the DSF and CFIR, but also analyzed data for emergent themes. |
| 27. | Software | What software, if applicable, was used to manage the data? | No software besides Excel was used to analyze data. |
| 28. | Participant checking | Did participants provide feedback on the findings? | No participants provided feedback on our findings. |
| Reporting | | | |
| 29. | Quotations presented | Were participant quotations presented to illustrate the themes / findings? Was each quotation identified? | Yes, quotes are presented throughout the study with identifiers attached. |
| 30. | Data and findings consistent | Was there consistency between the data presented and the findings? | Yes, data and findings are consistent. |
| 31. | Clarity of major themes | Were major themes clearly presented in the findings? | Major and minor themes are presented throughout the results. |
| 32. | Clarity of minor themes | Is there a description of diverse cases or discussion of minor themes? | Major and minor themes are presented throughout the results. |
| Source: Allison Tong, Peter Sainsbury, Jonathan Craig, Consolidated criteria for reporting qualitative research (COREQ): a 32-item checklist for interviews and focus groups, International Journal for Quality in Health Care, Volume 19, Issue 6, December 2007, Pages 349–357. | | | |
